# Silk protein nanofibers for highly efficient, eco-friendly, optically translucent, and multifunctional air filters

**DOI:** 10.1038/s41598-018-27917-w

**Published:** 2018-06-25

**Authors:** Kyungtaek Min, Sookyoung Kim, Sunghwan Kim

**Affiliations:** 1 Department of Energy Systems Research, Ajou University, Suwon, 16499 Republic of Korea; 2 Department of Nano-Optical Engineering, Korea Polytechnic University, Siheung, 15073 Republic of Korea; 3 Department of Physics, Ajou University, Suwon, 16499 Republic of Korea

## Abstract

New types of air filter technologies are being called because air pollution by particulate matters (PMs) and volatile organic compounds has raised serious concerns for public health. Conventional air filters have limited application and poor degradability and they become non-disposable wastes after use. Here, we report a highly efficient, eco-friendly, translucent, and multifunctional air purification filter that is highly effective for reducing air pollution, protecting the environment, and detecting hazardous chemical vapors encountered in everyday life. Uniform silk protein nanofibers were directly generated on a window screen by an electrospinning process. Optical properties (translucence and scattering) of the silk nanofibrous air filters (SNAFs) are advantageous for achieving viewability and controlling the room temperature. Air filtration efficiencies of the fabricated SNAFs could reach up to 90% and 97% for PMs with sizes under 2.5 and 10 μm, respectively, exceeding the performances of commercial semi-high-efficiency particulate air (semi-HEPA) filters. After use, the SNAFs could be naturally degraded. Furthermore, we demonstrate the ability of SNAFs impregnated with organic dyes to sense hazardous and volatile vapors encountered in everyday life.

## Introduction

Since the “Industrial Revolution,” human activities have caused a great deal of pollution. In particular, particulate matter (PM), the term used to describe a mixture of few-micron-sized solid particles and liquid droplets in air, has raised serious concerns with respect to public health owing to their small size and strong adhesion to hazardous chemicals^1,2^. It was reported that 3.45 million premature deaths was related to the inhalation of PM_2.5_ (fine PM with an aerodynamic diameter of 2.5 μm or less) in 2007 worldwide, and this number would be increased nowadays^3,4^. During respiration, PM_10_ (fine PM with a diameter of 10 μm or less) can accumulate in bronchioles through the airway^5,6^. More seriously, PM_2.5_ (fine PM with a diameter of 2.5 μm or less) can easily get through the human respiratory tract and reach the alveoli, inducing various short-term effects on mortality^5–7^. When the PM acts as an agent to transfer airborne hazardous chemicals and heavy metals directly to the deep organs, it can also cause adverse effects on human health^8,9^.

To deal with air pollution, the use of fibrous air filters has been a widely applied and reliable approach because of their low-cost and high efficiency, owing to their high surface-to-volume ratio and large porosity^10–12^. In particular, this approach is useful for protecting individual and residential housing, where a huge ventilation or central air conditioning system cannot be equipped. After use, however, conventional fibrous air filters that contain glass and chemically synthesized polymers (*i*.*e*. polyethylene (PE) and polypropylene (PP)) require additional disposal processes because of their poor degradability. Since these materials are not degraded naturally, their poor disposability is a serious social problem in various industries^13^. The used air filter becomes a waste with lots of captured pollutants or releases secondary pollutants during the disposal processes such as incineration or dissolution^14,15^. Additionally, the inflexible material traits restrict the sphere of application, except for air-filtration. To produce an eco-friendly and versatile air filter, it is necessary to explore new materials that could be naturally degradable and easily functionalized.

Electrospinning, an electrostatic fiber fabrication technique, has recently attracted increased interest owing to its versatility and potential application in diverse fields^16,17^. It enables low-cost and high throughput fabrication of polymeric nanofibers as well as serves as a means of controlling the material properties of nanofibers such as stiffness, fiber diameter, and porosity. The growing demand in bioapplication and environmental preservation necessitates the use of natural biopolymers including polysaccharides^18–20^, proteins^21–23^, and DNA^24,25^, because of their sustainability, eco-friendliness, and renewable traits based on their intrinsic properties such as biocompatibility and degradability. Moreover, in order to tailor the optical properties and functionalities of polymeric electrospun nanofibers, a variety of optically active dopants including organic dyes^26–28^, inorganic quantum dots^29–31^, and metal nanoparticles^32,33^ have been employed. Recently, protein-based electrospun nanofibers have been developed as biocompatible, disposable and highly efficient nanofibrous filters for air purification^12,34–37^. Nevertheless, silk fibroin (a natural protein from the *Bombyx mori* cocoon) can be a valuable candidate to generate functionalized and optically activated fibrous membranes^12,22,38^ due to its feasibility of developing versatile and eco-friendly air filters.

Here, we report a highly efficient, eco-friendly, optically translucent, and multifunctional air filter based on silk protein directly electrospun on a window screen, which can be utilized for highly efficient air filtration, improvement of indoor energy efficiency, and detection of hazardous vapors at the same time. Uniform silk nanofibers were successfully generated on a metal window screen. They showed translucency depending on the area density of the nanofibers. Nanoscale dimension of the silk nanofibrous air filter (SNAF) enables both high filtering efficiency for PM and low resistance to air flow, unattainable with air filters based on bulky micron-sized fibers. The prepared SNAF is eco-friendly; it can be degraded and removed without releasing pollutants. Nanofibers with ~300 nm diameter induce a relatively higher scattering in the visible range of wavelength than that in mid-infrared (mid-IR), and therefore, the SNAF on a window screen is useful for thermal management indoor. Additionally, the naturally extracted silk protein is doped with the natural organic dyes to generate eco-friendly and biocompatible fluorescent SNAFs. SNAFs functionalized with dyes display highly sensitive colorimetric and fluorescent chemosensing capabilities used for the detection of ammonia (NH_3_) and methanol (MeOH) vapors, respectively.

## Results and Discussions

**Figure 1:**
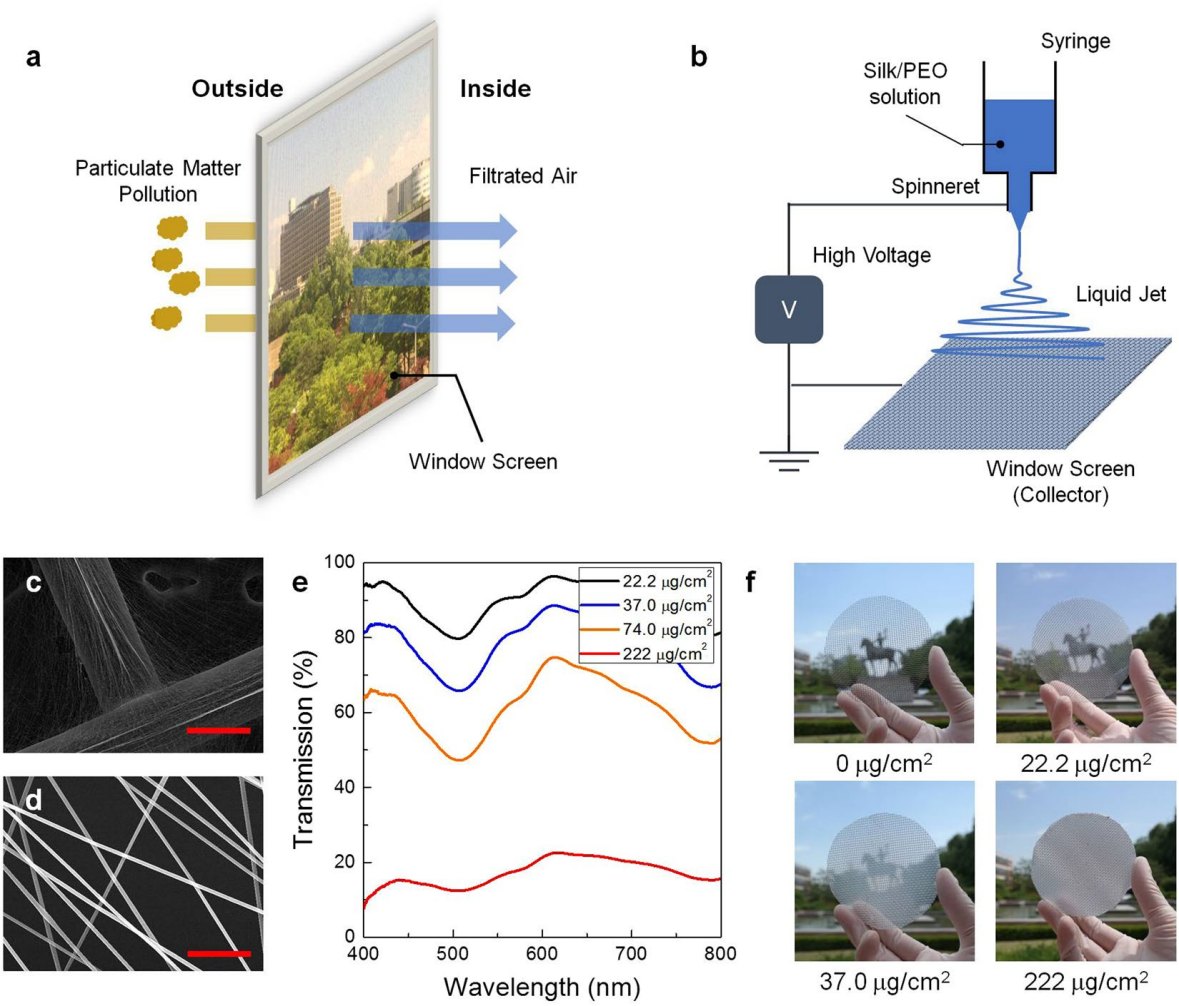
A high-performing, optically translucent silk nanofibrous air filter (SNAF). (**a**) Schematic illustrating a SNAF that can be mounted on a window. (**b**) A schematic of electrospinning process used for the deposition of silk nanofibers on a window screen. (**c**) A SEM image of the silk nanofibers densely integrated with the metal wire. (**d**) A SEM image of the silk nanofibers uniformly distributed in a non-metal region. Scale bars represent 250 μm for (**c**) and 6 μm for (**d**). (**e**) Transmittance spectra of the SNAFs with area densities of 22.2, 37.0, 74.0, and 222 μg/cm^2^. (**f**) Photographs of the translucent SNAFs with nanofiber area densities of 0, 22.2, 37.0, 222 μg/cm^2^ demonstrating the change in their translucency.

Figure 1a shows the scheme for the generation of SNAF on a window screen. The silk protein-based nanofibrous air filter can efficiently block the fine PM entering from outside and can still have visibility. A mixture of an aqueous silk solution and polyethylene oxide (PEO) was prepared for the electrospinning process. PEO was used to prevent embrittlement of silk nanofibers due to the formation of the water-insoluble *β*-sheet^22^. To generate the nanofibrous membrane, the silk/PEO solution was injected into a steel spinneret and then electrically charged with different electric voltages applied between the spinneret and the bottom metal window screen (Fig. 1b). As the injected jet dried and got elongated by electric repulsion during the whipping process, the elongated silk nanofibers were collected on the window screen made up of a network of metal wires with a diameter of 300 μm. A scanning electron microscopy (SEM) image of the material (Fig. 1c) shows that the electrospun nanofibers had been densely collected on the metal wire network area. However, despite a lower density, uniform nanofibers with a diameter of 300 nm were also evenly distributed and formed a membrane for air filtration in the non-metal region (Fig. 1d).

Owing to the thinness and the high porosity of the nanofibrous membranes, SNAFs could be rendered translucent depending on the electrospinning time and the area density of nanofibers. The mass of silk fibers generated on a 1 cm^2^ area of the window screen per minute was found to be 7.4 μg. Figure 1e shows the optical transmittances of the SNAFs with various area densities. The SNAFs with area densities of 22.2, 37.0, and 74.0 μg/cm^2^ had transmittances over 80, 60, and 40% (at 500 nm), respectively, in the visible spectral range, enabling visibility through the window (Fig. 1f). In contrast, the SNAF was almost opaque at the density of 222 μg/cm^2^. Interestingly, a spectral dip occurs at 507 nm in the transmittance spectrum. Finite-difference time-domain (FDTD) simulation indicates that this originates from light scattering by randomly distributed nanofibers with a diameter of 300 nm, and this can be tuned by changing the diameter of the nanofiber (Supplementary Fig. S1).

**Figure 2:**
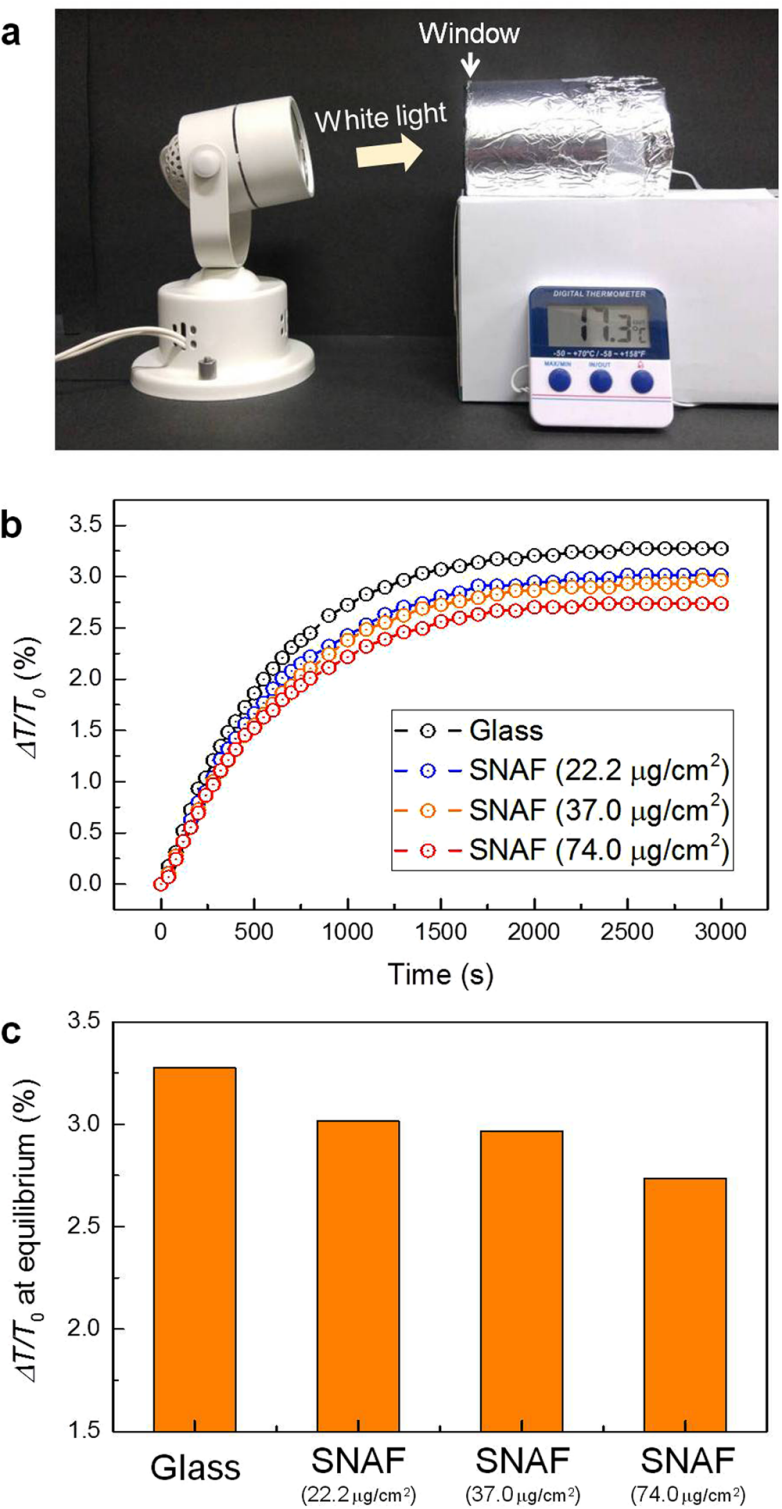
Thermal management using the SNAF. (**a**) A photograph showing the proof-of-concept experiment setup used to investigate radiative heating and cooling effects. A transparent glass plate or the translucent SNAF was mounted at the entrance of the room. (**b**) Rate of increase in temperature (*ΔT/T*_0_) in the closed room, where *ΔT* is change of the temperature and *T*_*0*_ is the initial temperature. (**c**) Measured *ΔT/T*_0_ at equilibrium states of the temperature.

Using the SNAF, the intensity of visible light entering the room can be controlled. The incident visible light radiated from the sun, a blackbody with a temperature of 6600 K, increases the temperature of the earth’s atmosphere upon its absorption. Subsequently, the earth emits mid-IR radiation of ~10 μm wavelength (radiative cooling), corresponding to the emission from a blackbody with 288 K^39,40^. Since the nanoscale diameter of silk fibers is much smaller than the mid-IR wavelength, mid-IR scattering by the nanofiber is negligible, and therefore, the SNAF is expected to be effective for radiative cooling. As a proof-of-concept, a closed room with an window was heated using white light and the temperature was measured to investigate the radiative cooling effect via visible radiation heating and mid-IR radiation cooling (Fig. 2a). A transparent glass plate and translucent SNAF with area densities of 22.2, 37.0, and 74.0 μg/cm^2^ were mounted on the window. As shown in Fig. 2b, the temperature increased gradually from the initial temperature of 288 K and reached an equilibrated temperature in 3000 s. Interestingly, the equilibrium temperature in the presence of SNAF was found to be 0.3–0.4% (~1 K) less than that when only the glass plate was used (Fig. 2c). This demonstrates that the SNAF-loaded window screen is attractive for improving the indoor energy efficiency.

**Figure 3:**
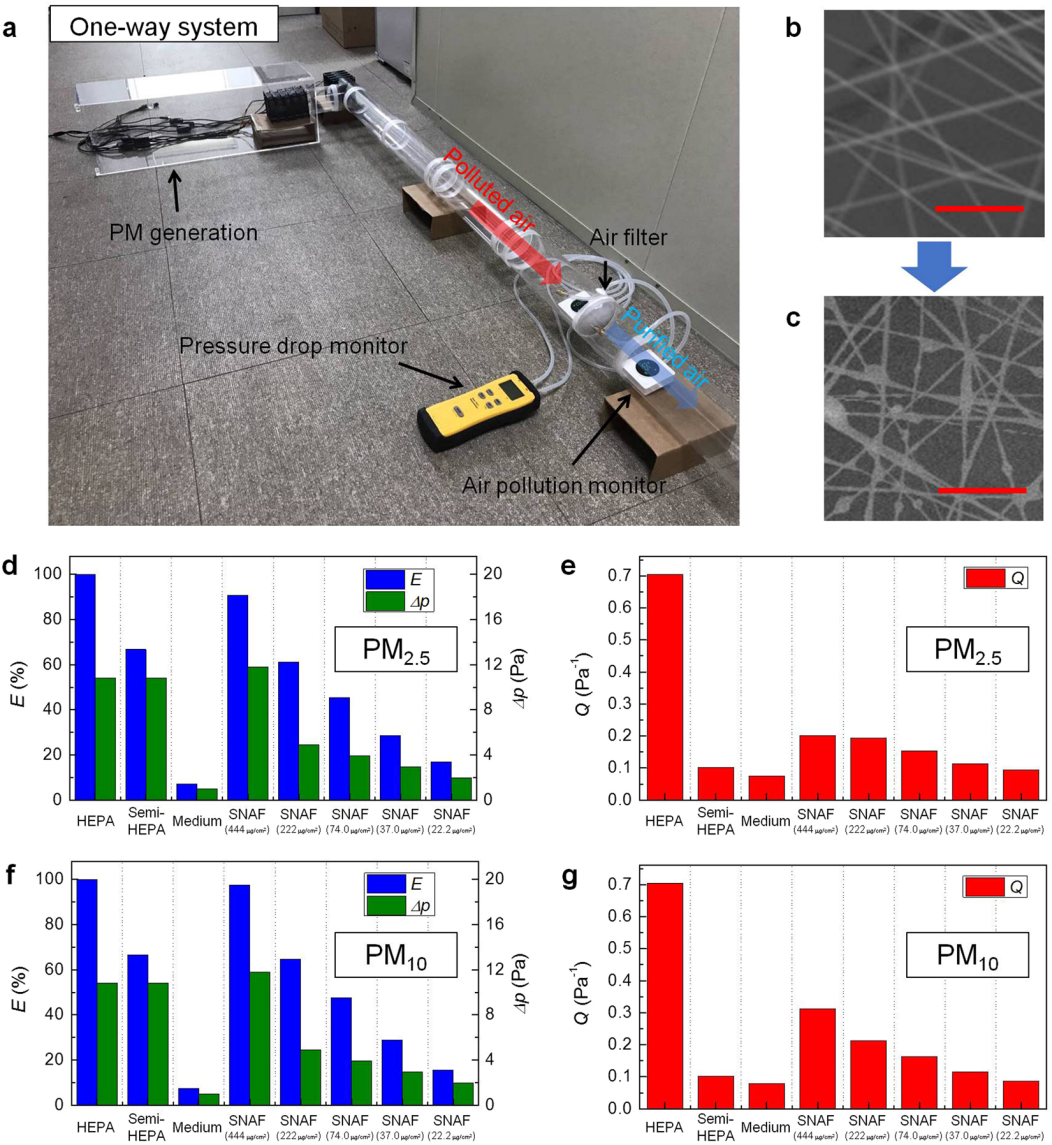
Filtration performance of the SNAFs. (**a**) A photograph of the “one-way” setup used to investigate the filtration efficiencies and pressure drops of the air filters. SEM images of the nanofibers with a density of 22.2 μg/cm^2^ (**b**) before and (**c**) after the air filtration process. Scale bars represent 10 μm. (**d**) Filtration efficiencies (blue bars), pressure drops (green bars), and (**e**) quality factors (red bars) of various kinds of air filters for PM_2.5_ filtration. (**f**) Filtration efficiencies (blue bars), pressure drops (green bars), and (**g**) quality factors (red bars) of the air filters for PM_10_ filtration.

Filtration performance, determined by the PM filtration efficiency and resistance of air flow, of the SNAFs with different area densities were investigated and compared to those of commercially available air filters. As shown in Fig. 3a, PM filtration by air filters could be demonstrated using a simple “one-way” setup. Incenses were burned to generate the PM. The polluted air was flown through a circular acrylic pipe and then purified once by the air filter. The purified air was released outside after filtration. The air flow was controlled using electrically driven air fans. To estimate the filtration efficiency and pressure drop, the PM concentration and air pressure were monitored on the frontside and backside of the air filter. Figure 3b and c show the SEM images of the SNAF with an area density of 22.2 μg/cm^2^ before and after air filtering. PMs were effectively captured by the silk nanofibers when the polluted air was passed through the SNAF, confirming the high adhesion properties of silk fibers^12^. The filtration efficiency of the SNAF was estimated by measuring the concentration of PMs in the flowing air before and after filtering. Commercial high-efficiency particulate air (HEPA) (H13 grade), semi-HEPA (E10 grade), and medium (M5 grade) fibrous filters with fiber diameters of 3, 10, and 20 μm, respectively (Supplementary Fig. S2), were chosen for comparison. For the filtration of PM_2.5_, as shown in Fig. 3d, the filtration efficiency of the SNAF could be improved to ~90% by increasing the area density of nanofibers spun on the window screen. Note that, the higher the density of nanofibers is, the better the filtration efficiency of the filter is. However, the filter becomes opaque at high density of nanofibers. In general, nanofibers are more effective in capturing PMs than microfibers. The filtration efficiency of the SNAF is superior to those of commercial semi-HEPA and medium filters. Although the SNAFs show slightly lower efficiency than that of the H13 grade HEPA filter, which is the highest rating to evaluate the air filter, it still has an attractive functional feature that would be useful in everyday life, as would be discussed.

Besides the filtration efficiency, preserving the air flow is another important factor to assess the performance of an air filter. The overall performance of the air filters, considering both their filtration efficiency and pressure drop, can be evaluated by the quality factor *Q* defined as,

$$Q=\frac{-{\rm{ln}}(1-E)}{\triangle p}$$

where, *E* is the filtration efficiency and *Δp* is the pressure drop^11^. For the PM_2.5_ filtration performance, the *Q* values of the SNAF for all the densities studied are remarkably better than those of the commercial semi-HEPA and medium filters, and are comparable to those of other reported nanofibrous filters (Fig. 3e)^11,12^. This indicates that SNAF could show satisfactory filtration performance even when the SNAF layer is thinner and hence optically translucent. Further, as expected, the PM_10_ filtration performances for larger dust particles (Fig. 3f,g) are better than the overall PM_2.5_ filtration performance (up to *E* = 97% and *Q* = 0.3 at the density of 444 μg/cm^2^).

**Figure 4:**
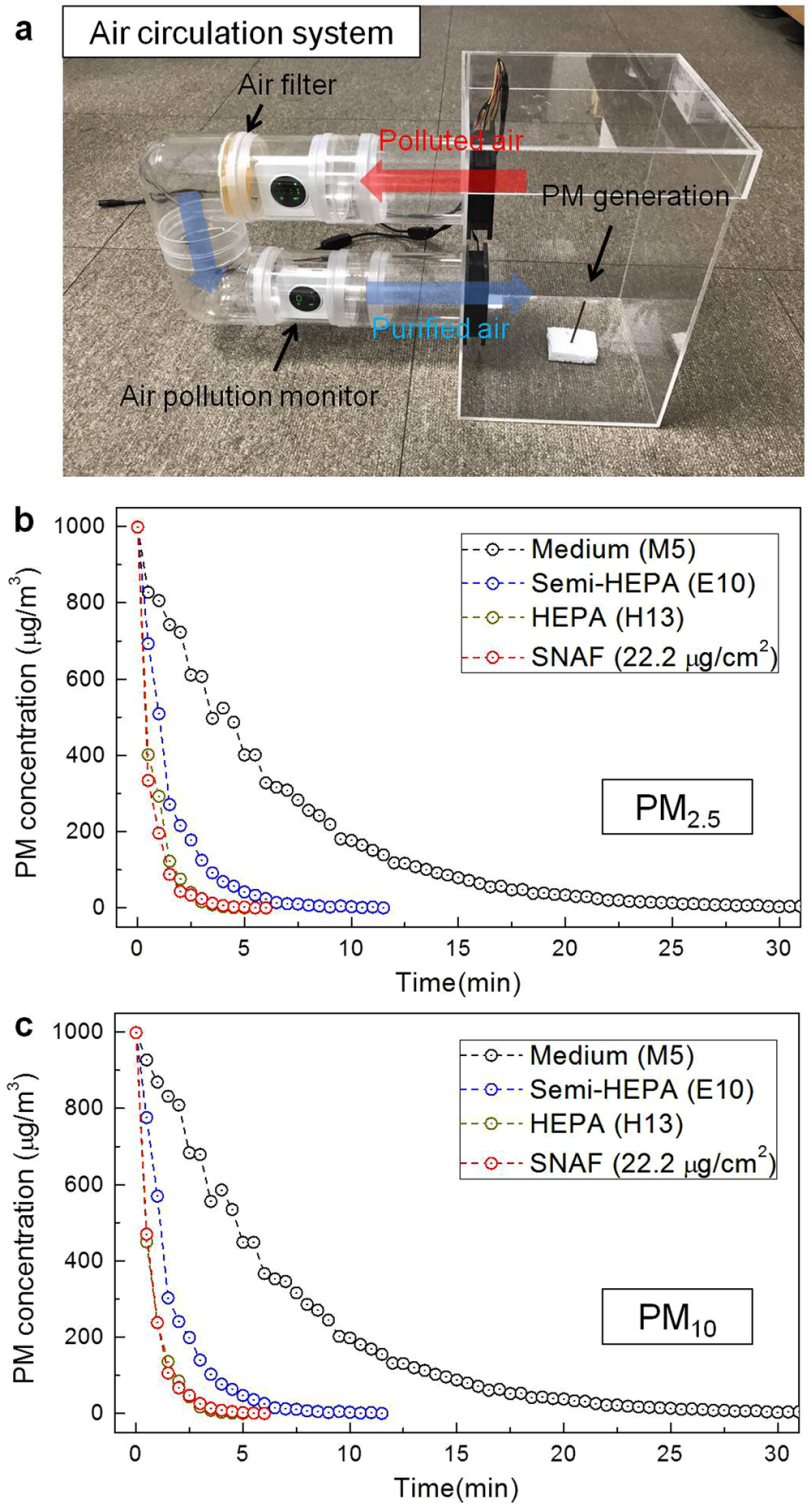
Filtration performance of the SNAFs in the circulation system. (**a**) A photograph of the “circulation” setup used to investigate the overall filtration performances of the air filters. Changes in the (**b**) PM_2.5_ and (**c**) PM_10_ concentrations in the air circulation systems upon adapting HEPA (H13), semi-HEPA (E10), medium (M5) and the SNAF (with 22.2 μg/cm^2^ density), respectively.

Next, we applied the SNAF to an air circulation system and investigated the decay rates of the PM concentration to evaluate their overall performance in the circulating environment (Fig. 4a). Polluted air was circulated by an air fan in a closed loop, and the air was purified by the air filter installed in the passage. For HEPA, semi-HEPA, and medium fibrous filters, and silk nanofibrous (22.2 μg/cm^2^) filters, times taken to remove all PM_2.5_ at the high mass concentration of 1000 μg/m^3^ from air are 5, 12, 30, and 6 min, respectively (Fig. 4b). Clearly, the better the filter performance (the higher the *Q*), the less time it takes to completely remove the PM. For PM_10_, all the filters exhibited similar performances since all the filters were designed to efficiently remove PMs with sub-micron sizes. Interestingly, SNAFs for all densities showed superior filtering capabilities, comparable to that of the HEPA filter (Supplementary Fig. S3). We estimate that the *Q* values of the SNAFs can be increased under increased pressure of the air flow. In addition, repeatability tests were performed to investigate the decline in the performance. The low-density SNAF (22.2 μg/cm^2^) exhibited decline of 33% (time taken to remove all PM) after 10 iterations, whereas the SNAFs with densities over 74.0 μg/cm^2^ exhibited no decline (Supplementary Fig. S4a). Supplementary Fig. S4b shows that the reduction in the performance of the SNAF with a density of 22.2 μg/cm^2^ is accompanied by a reduction in the optical transmission (17%), indicating that the PMs attached to nanofibers stem the air flow and induce additional scattering and absorption.

**Figure 5:**
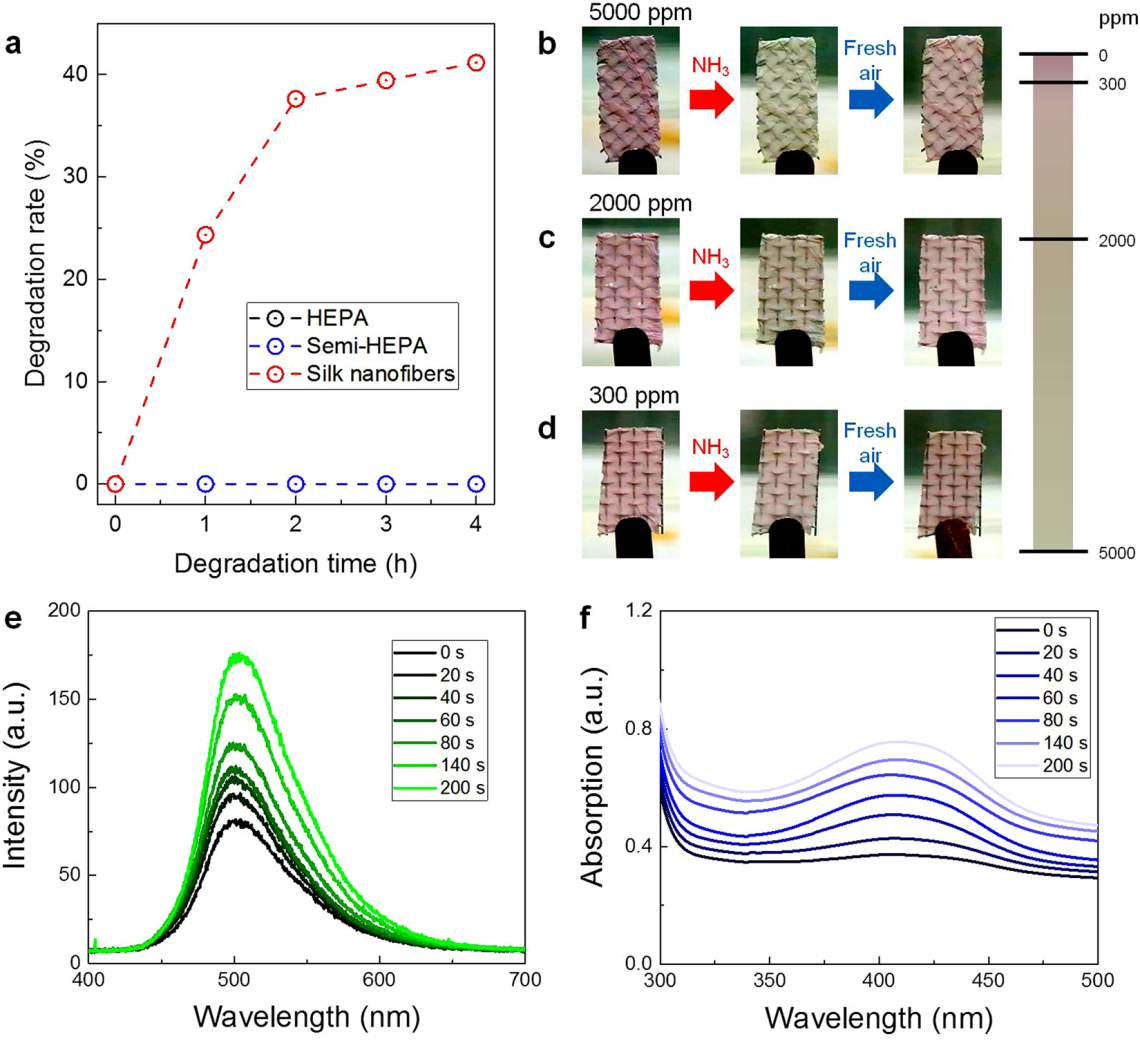
Multifunctionality of the SNAF. (**a**) Degradability of the HEPA, semi-HEPA, and silk nanofiber air filters. (**b–d**) Results of the NH_3_ vapor sensing experiment at lethal concentrations (red arrows). Changes in the external color of the anthocyanin-doped silk nanofiber membranes at (**b**) 5000, (**c**) 2000, and (**d**) 300 ppm of NH_3_. The responses are reversible (blue arrows). A color bar represents the concentration-dependence of the color changes of the silk nanofibers. (**e**,**f**) Results of the MeOH vapor sensing experiment. Time-dependent changes in the (**e**) fluorescence and (**f**) absorption spectra of the coumarin-doped silk nanofibers exposed to MeOH fume.

Natural degradability of the used material is important from the aspect of environmental preservation. Glass, PE and PP, which are commonly used to fabricate fibrous filters, are non-biodegradable, and numerous toxic substances are generated during their disposal by processes such as incineration or dissolution. In contrast, our SNAF based on natural silk protein is eco-friendly, degradable, and causes no environmental pollution. We investigated the biodegradability of the commercial filters and silk nanofibers using enzymes (see *Methods* for more details)^41,42^. Silk nanofibers exhibited a high degradation rate whereas the other commercial filters (composed with glass, PE and PP) did not (Fig. 5a). This indicates that polluting processes in the disposal of used SNAFs can be significantly reduced. The degradation rate of the SNAFs can be also controlled by tuning the crystallinity, or composition of the primary structure (random coil) and the secondary structure (*α*-helix and *β*-sheet), of the silk protein^38^.

In addition to its use as an efficient air filter, the SNAF can also be applied as a highly sensitive chemosensor after doping functional dyes. The high surface-to-volume ratio and the polymeric nature of the silk nanofibers are advantageous for their utilization as highly sensitive colorimetric and fluorescent vapor sensors^38^. Anthocyanin-doped SNAF was prepared on a window screen to detect NH_3_, a noxious and volatile gas in daily life and industries. Anthocyanin, a natural dye extracted from purple sweet potato, is suitable to detect the NH_3_ vapor due to the pH dependence of its external color^43^. As shown in Fig. 5b–d, the SNAF NH_3_ chemosensor immediately provided visual responses (color changes from purple to yellow) upon detecting the NH_3_ gas at different concentrations (300, 2000, and 5000 ppm). When the anthocyanin-doped SNAF was exposed to the NH_3_ vapor, the equilibrium of the anthocyanin molecules was shifted from the purple quinoidal anhydrobase (pH ~ 7) to the yellow chalcone (pH > 8)^44^. The concentrations used were determined because the lowest lethal concentration (LC_Lo,5min_, for human) and respiratory rate (RD_50_, for mice) of NH_3_ gas, assessed by the US Center for Disease Control and Prevention, are 5000 and 300 ppm, respectively^45^. Additionally, the color responses are reversible upon exposure to fresh air (blue arrow). Increasing the concentration of NH_3_ gas resulted in more drastic color changes of the nanofibers.

MeOH vapor was also chosen as an analyte for the chemosensing since it is also widely encountered in daily life and harmful for human health. As a probe for the MeOH detection, coumarin, a natural organic chemical compound found in many plants and sensitive to the MeOH vapor^46^, was applied to the SNAF. Figure 5e shows the fluorescent response of the coumarin-doped silk nanofibers exposed to MeOH fume. As the SNAFs were exposed to the MeOH fume, the fluorescence intensity of the dye gradually increased. When the chemical reactions were saturated (200 s), the fluorescence intensity of the coumarin dye approximately doubled compared to the initial value. As shown in Fig. 5f, the increase in the fluorescence of the coumarin-doped nanofibers originates from the enhancement of the absorption of the coumarin dye exposed to MeOH vapor.

## Conclusion

In conclusion, we demonstrated that an eco-friendly, highly efficient, and multifunctional SNAF could be applied as a translucent window screen with air-filtering and chemosensing functions. Uniform and large-area silk nanofibrous membranes were successfully loaded on the metal wire window screen via electrospinning. Owing to the transparency of the silk nanofiber network, it is suitable for installing on windows toward viewability and controlling the room temperature. The SNAFs efficiently captured fine particles with sizes under 2.5 μm (PM_2.5_) and 10 μm (PM_10_) entering in from the outdoor environment. The biodegradability of the silk nanofibers aids in overcoming the limitation of the conventional fibrous filters which release non-decaying materials during their disposal. In addition, SNAFs with organic dye probes were applied as highly sensitive chemosensors for detecting hazardous vapors encountered in daily life. Our results strongly suggest that electrospun silk nanofibers are an outstanding material that can be applied in environmental and biomaterial sciences.

## Methods

### Preparation of aqueous silk solution

Cocoons of *Bombyx mori* caterpillars were boiled for 30 min in an aqueous solution of 0.02 M Na_2_CO_3_ to remove sericin proteins. Then, the extracted silk fibroin was rinsed thrice with distilled water for 20 min and dried for 24 h. The dried silk fibroin was dissolved in a 9.3 M LiBr solution and incubated in an oven at 60 °C for 4 h. This solution was dialyzed against distilled water using a dialysis membrane (Cellu-Sep T1, MWCO 3.5 K, Membrane Filtration Products) at room temperature for 48 h. The obtained solution was centrifuged twice for 20 min at −1 °C and 9,000 rpm to remove impurities. The concentration of the final silk fibroin aqueous solution was approximately 8 wt%.

### Electrospinning process for generating silk nanofibers

The addition of poly(ethylene oxide) (PEO, M_v_ ~900,000, Sigma-Aldrich) to silk solutions generated a mixture with viscosity and surface tension suitable for electrospinning. The base solution was prepared by mixing a 5 wt% PEO solution and the prepared silk solution at the ratio of 1:1. The silk/PEO solution was loaded into a syringe and the syringe was mounted on the electrospinning machine. A metal window screen was used as the collector. The distance between the tip and the collector was 20 cm, and the flow rate of the bio-ink was set to 10 μL/min. An electric potential of 10 kV was applied between the 21 G nozzle tip and collector during electrospinning for durations between 3 min and 2 h. To generate dye-doped silk nanofibers, an anthocyanin dye (Natural Anthocyanin Dye, MSC Co.) extracted from purple sweet potato was mixed with the base silk/PEO solution at the ratio of 1 wt% for the ammonia sensor, and a coumarin dye (Coumarin 481, Exciton) was mixed with the base solution at the ratio of 0.14 wt% for the MeOH sensor.

### Room-temperature control experiments

For the preparation of a closed cylindrical room, the inside of an acrylic pipe was wrapped with black paper that could block visible light and the exterior was wrapped with an aluminum foil to cut off the heat from the outside except from the main white light source. A transparent glass plate or silk nanofibrous window screens were placed at the entrance-side of the closed room. The closed room was irradiated by white light emitted from a 20-W dichroic lamp (44860WFL, OSRAM) and the internal temperature was measured with a thermometer (RT-803, Daekwang, Inc.).

### Evaluation of air filtration performances

In the one-way system, a PM generation chamber with a size of 20 cm × 20 cm × 65 cm was prepared and connected with an acrylic pipe with a diameter of 8 cm and a length of 160 cm to allow dust particles to flow in a single direction. An electrically driven air fan was installed at the outlet of the PM generation room to induce air flow. The air filters were placed at a distance of 130 cm from the PM generation room for air purification. In the circulation system, a PM generation room with the size of 15 cm × 15 cm × 30 cm was prepared, the input and output port of which were connected with an U-shaped pipe with a diameter of 8 cm. An air fan was installed at the outlet of the PM generation room to induce air circulation. Incense was burned to generate polluted air, PMs of which were composed of 85% PM_2.5_ and 15% PM_10_. Air pollution was monitored using a commercial fine dust concentration meter (Laser PM Detector, Shenzhen Fineair Smart Life Technology Co.), and the air pressure was measured with a commercial manometer (SDMN5, Fieldpiece). To compare the performance of the conventional filters and SNAFs, we prepared commercial HEPA filter (Filtertec, H13), semi-HEPA filter (Plus Minus Zero, E10), and medium filter (Oclaire, M5), which were composed with PP, glass/PP, and PE/PP, respectively.

### Optical measurements

Optical properties of the silk nanofibers were investigated using a visible/near-infrared fiber-optic spectrometer (USB-2000, Ocean Optics). For obtaining the reflectance spectra, a 1 × 2 fiber coupler enabled white light to be fed to the nanofibers, while simultaneously recording the reflected signal. The reflected optical signal was directly fed into the spectrometer through a fiber. Another spectrometer (V-670, Jasco Inc.) was used to measure the transmittance spectra of the samples and the absorbance spectra was estimated using the relationship, *A* = 1 − *R* − *T* where *A*, *R*, and *T* denote the absorbance, reflectance, and transmittance, respectively. The coumarin-doped nanofibers were optically pumped by a frequency-tripled 355-nm Nd:YAG picosecond laser (PL2210A, Ekspla) for obtaining their fluorescence spectra. SEM images were obtained (S-4800, Hitachi) to observe the morphology of silk nanofibers.

### Preparation of vaporized NH3 and concentration control

To prepare the NH_3_ vapor environment on a ppm scale, liquid NH_3_ was dropped into a beaker using a micropipette. After sealing the beaker, it was incubated for 1 h at room temperature to completely vaporize the ammonia solution. For achieving a concentration of 1 ppm (v/v), the ratio of the volume of the NH_3_ vapor to that of the beaker was adjusted to 1 μL/L. By evaporating 1.31 μg of liquid NH_3_, 1 μL of NH_3_ vapor could be obtained, which was estimated from the molar mass to molar volume ratio at room temperature.

## Electronic supplementary material

Supporting Information
