# Supplementary material for: Silk protein nanofibers for highly efficient, eco-friendly, optically translucent, and multifunctional air filters
Source: Sci Rep. 2018 Jun 25;8:9598. doi: 10.1038/s41598-018-27917-w (PMC6018553; doi:10.1038/s41598-018-27917-w)
Supplement: Supplementary file 1 — Supporting Information [file 41598_2018_27917_MOESM1_ESM.docx]

**Supplementary Information**

Silk protein nanofibers for highly efficient, eco-friendly, optically translucent, and multifunctional air filters

Kyungtaek Min^1,2,†^, Sookyoung Kim^1,†^ & Sunghwan Kim^1,3,*^

^1^Department of Energy Systems Research, Ajou University, Suwon 16499, Republic of Korea

^2^Department of Nano-Optical Engineering, Korea Polytechnic University, Siheung 15073, Republic of Korea

^3^Department of Physics, Ajou University, Suwon 16499, Republic of Korea

^*^[To whom correspondence should be addressed. E-mail: sunghwankim@ajou.ac.kr](mailto:sunghwankim@ajou.ac.kr)

^†^These authors contributed equally to this work.

**Simulated transmittance spectra of the silk nanofiber membranes**

**Figure S1.** (a) A scheme showing randomly distributed silk nanofibers with a radius *r*. (b) The transmittance spectra of the silk nanofiber membranes obtained by finite-difference time-domain (FDTD) simulation.

**SEM images of commercial air filters**

**Figure S2.** Scanning electron microscopy images of commercial air filters: (a) HEPA filter, (b) semi-HEPA filter, and (c) medium filter. Scale bars represent 100 μm.

**Performance of silk nanofibrous air filters (SNAFs) with different area densities**

**Figure S3.** Decreases in the (a) PM_2.5_ and (b) PM_10_ concentrations in the air circulation systems adapting SNAFs with area densities of 7.4, 22.2, 37.0, and 74.0 μg/cm^2^, respectively. PM_2.5_ and PM_10_ represent particulate matter with diameters of particles less than 2.5 and 10 μm, respectively.

**Deterioration of the performance of the low-density SNAF (22.0** μ**g/cm^2^) after repeated used**

**Figure S4.** Performance decline of the low-density SNAF (22.0 μg/cm^2^) by N iterations of the PM_2.5_ filtration process. (a) Filtration times required to purify air from 1000 to 0 ppm of PM_2.5_. (b) Change in the transmission spectrum of the low-density SNAF.
